# Risk profiles of referred chronic kidney disease patients in a tertiary nephrology centre

**DOI:** 10.12669/pjms.321.8214

**Published:** 2016

**Authors:** Christopher Thiam Seong Lim, Raihan R, Mohd Zharif Fikri A.S.

**Affiliations:** 1 Christopher Thiam Seong Lim, Department of Medicine, Faculty of Medicine and Health Sciences, Universiti Putra Malaysia, Serdang, Malaysia; 2 Raihan R, Department of Medicine, Faculty of Medicine and Health Sciences, Universiti Putra Malaysia, Serdang, Malaysia; 3 Mohd Zharif Fikri A.S, Department of Medicine, Faculty of Medicine and Health Sciences, Universiti Putra Malaysia, Serdang, Malaysia

**Keywords:** Chronic kidney diseases, Proteinuria, Co-morbidities, Glomerular fitration rate

## Abstract

**Objective:**

To determine the risk profile of chronic kidney disease (CKD) patients.

**Methods:**

This is an observational cross sectional study involving 130 patients for which data was collected prospectively.. Sixty five subjects with an estimated glomerular filtration rate (eGFR) below 120 ml/min/1.73 m^2^ were recruited via random sampling technique from nephrology clinic in a tertiary nephrology referral center. Medical history, demographic data, urinary and serum biochemistry tests were obtained.

**Results:**

Most of the CKD patients who were referred to the nephrology clinic were asymptomatic. The most common laboratory abnormalities noted among CKD patients were proteinuria, anaemia and hyperkalaemia. Advancing age, pre-existing morbidities such as diabetes mellitus, hypertension and overweight are characteristics that are strongly associated with the referred CKD patients.

**Conclusions:**

Most of the referred CKD patients are in advanced age with diabetes, hypertension and overweight. Majority of the CKD remained asymptomatic despite in the advance stage of CKD. This strongly calls for cost effective screening for population at risk.

## INTRODUCTION

Chronic kidney disease (CKD) is a major health problem as one of the non-communicable diseases. The prevalence of chronic kidney disease (CKD) is estimated at > 500 million individuals globally and this is partly due to increased prevalence of hypertension, diabetes and metabolic syndrome.^1^ A main challenge for targeting chronic kidney disease (CKD) is the heterogeneity of its causes, co-morbidities and outcomes. In a prevalence study done in Malaysia, the prevalence of CKD was 9.07% with only 4% of the respondents were aware of their diagnosis.^2^ Patients under nephrological care represent an important reference population, but knowledge about their characteristics is limited. As CKD is associated with increased cardiovascular mortality and a loss of disability-adjusted life years, 3 we undertook a prospective study of patients with CKD in a tertiary referral center to determine the risk profile of referred CKD patients.

## METHODS

This is a single hospital-based observational, cross sectional study. All the patients who were diagnosed as CKD were randomly selected from Serdang Hospital nephrology clinic based on pre determined randomized technique of recruitment of one patient per every three patients who attended the clinic to a sample of size of 65. Patients included were 18 years of age and above and diagnosed with Chronic Kidney Disease of stage 1 (GFR: >90 mL/minute/1.73m^2^; to stage 5 (GFR: <15 mL/minute/1.73m^2^) as per K/DOQI clinical practice guidelines.^4^ Patients who were below 18 years of age or had acute kidney injury were excluded. The control group was from the adjacent medical specialist clinic and they were all above 18 years of age with preclinical kidney disease with eGFR above 60 ml/min per 1.73 m2.

Demographic data such as age, gender, ethnicity, height, body mass index, aetiology of CKD, co-morbidities diabetes, hypertenson, cerebral vascular event and laboratory data such as blood pressure, creatinine, haemoglobin, HbA1C, serum calcium, serum phosphate, status of hepatitis B and hepatitis C, GFR, presence or absence of proteinuria and signs and symptoms related to CKD were collected.

The control group of patient, were recruited from the adjacent medical specialist clinic using the same sampling method as described earlier.

This study was non-interventional and did not influence the nature of treatment received by the subjects. Institutional review board (IRB) approval was obtained and either the patients or patients’ closest available next-of-kin were approached in all cases for informed consent using IRB-approved informed consent form. This was an investigator-initiated study.

All data was analysed using SPSS version19. Mean and standard deviation were used to describe continuous data while categorical data are described by using percentage. Confidence interval is set as 95% and Standard p value of p<0.05 is set for all significant levels. The normality of continuous data was tested using Kolmogorov-Smirnov test. The significant value was p ≤ 0.05. Binomial logistic regression is used to analyze the characteristic of referred CKD patients.

## RESULTS

A total of 130 patients were included into the study. The social-demographic data of the studied patients is shown in Table-I. The most common sign of the referred CKD patient was the presence of macroalbuminuria (70.8%), followed by hyperkalaemia (20%) and normochromic normocytic anaemia (83%).

**Table-I:** Social-demographic characteristic.

Category	Referred CKD (65 patients)	Control CKD (65 patients)
*Age (years)*		
<55	19	42*
>55	46	NIH23*
*Gender*		
Male	33	26
*Ethnicity*		
Malay	33	41
Non Malay	32	24

* Denotes significance at P < 0.05.

The mean GFR in the CKD referred group was 20 ± 15 ml/min/1.73m^2^ whereas the control group has 103 ± 46 ml/min/1.73m^2^. In the referred CKD group, 2% were with CKD 1, 20% with CKD 3, 32% with CKD 4 and 46% with CKD 5 satge of renal failure. Most of our patients were asymptomatic however 7.7% complained of lethargy, 7.7% of poor appetite, 12.3% had symptoms of fluid overload and only 1.5% had itchiness.

From the bivariate analaysis, there is significant association between CKD with the presence of underlying disease such as diabetes, hypertension and the manifestation of proteinuria. Significant laboratory markers apart from rasied serum creatinine and decreased GFR were the presence of hyperkalaemia, and anaemia (Table-II).

**Table-II:** Correlation of major co-morbidities with referred CKD status.

Co-morbidities	Referred patients				Total	Value		Confidence interval	

	Yes	N %	No	N %		Z	p-value	upper	lower
*DM*									
Yes	51	(63.8)	29	(36)	80	15.730	0.001*	2.099	9.741
No	14	(28.0)	36	(72)	50				
*HPT*									
Yes	54	(62.1)	43	(37.9)	87	15.325	0.001*	2.117	10.706
No	11	(25.6)	32	(74.4)	43				
*Proteinuria*									
Yes	46	(92.0)	4	(8.0)	50	41.068	0.000*	0.013	0.128
No	19	(31.7)	41	(68.3)	60				

Laboratory values	Mean±SD		Mean±SD		No	T	p – value	Upper	Lower

Creatinine	472.7±297		77.0±29.0		65/65	10.248	0.000^	36.9	3.8
GFR	20±15.1		103±46.1		65/65	-13.73	0.000^	1.8	6.3
Haemoglobin	10.6±1.6		12.3±2.3		65/65	-4.80	0.000^	1.6	0.2
Potassium	4.9±2.1		4.1±0.5		65/65	7.0	0.000^	0.6	0.5

DM: Diabetes Mellitus, HPT: Hypertension, GFR: Glomerular Filtration Rate

* Chi-square, p<0.05 denotes significance

^ T test, P <0.05 denotes significance.

Parameters like gender, ethnicity, serum glucose, calcium, phosphate, hepatitis serology, sodium level and clinic blood pressure readings were similar in referred and nonreferred group. Logistic regression analysis showed that typical profile of a referred CKD patient is that they are of older age group with features of hypertension and higher body weight than the control. The most common laboratory features were proteinuria and anaemia (Table-III).

**Table-III:** Logistic regression showing factors associated with referred CKD patients.

	B	S.E.	Wald	df	Sig.	Exp(B)
Ethnicity	-.027	.971	.001	1	.978	.973
Age	-.088	.044	4.014	1	.045*	.916
Weight	-.114	.049	5.556	1	.018*	.892
Diabetes	.483	1.247	.150	1	.698	1.621
Hypertension	1.881	1.055	3.183	1	.074	6.563
Proteinuria	4.701	1.611	8.511	1	.004*	110.069
HbA1C	.428	.261	2.680	1	.102	1.533
Heamoglobin	.806	.325	6.137	1	.013*	2.238
Potassium	-1.471	.867	2.876	1	.090	.230
Constant	1.048	5.546	.036	1	.850	2.852

* Denotes significance at P<0.05.

## DISCUSSION

CKD is an important cause of death and disability but its awareness is low among patients and health care professionals. The burden of CKD is rising in Malaysia, as shown by increases in attributable deaths and incidence and prevalence of ESRF.^2^ In order to retard the progression of CKD at early stage, awareness and use of cost effective screening methods are needed to enable timely diagnosis and management.

Our hospital is a fully subsidized hospital where the patients only require to pay less than USD2 per visit which includes laboratory tests and medications. Senior citizens, those who are above 65 year of age, receive treatment free. Despite that, the majority of CKD patients who attended our referral clinic were already in the advance stages of CKD despite our department policy of accepting CKD of all stages. Most of the referred CKD patients were symptomatic. This highlights that there is a need to create high degree of awareness among the primary care physicians to perform appropriate health screening for at risk population.

In our study population, advanced age was found to be associated with CKD. The cause of age-related increases in renal fibrosis, which leads to glomerulosclerosis, interstitial fibrosis, tubular atrophy, vascular sclerosis, and loss of renal function, is poorly understood. However, in animal models, collagen seems to accumulate with age in the glomerulus, peritubular capillaries and tubulointerstitium because of increased transcription of the gene encoding type III collagen.^5^

Amongst the referred patients, there were 63.8% diabetics and 62.1% hypertensive. Similar to other countries, both diabetes and hypertension are among the growing non-communicable disease burden.^1^ Although diabetes and hypertension appeared to be signficantly associated with CKD in bivariate analysis, their association were diminished after logistic regression analysis. This is likely due to the high number of turn up of diabetic and hypertensive patients in the medical specialist clinic for other micro and macro complications of diabetes and hypertension.

The high correlation of proteinuria in CKD reinforce the fact that proteinuria is a reliable marker for CKD. Our study has shown that the risk of having CKD is increased ten folds with proteinuria regardless of the aetiology of CKD. Proteinuria is a proven marker of kidney injury and chronic kidney disease.^6^,^7^ Presence and severity of proteinuria should be ascertained managing the population at risk. Renal protection via the angiotensin-converting enzyme inhibitors should be then prescribed to slow progression of nephropathy.^8^

Our study concurred with two studies done in USA which showed targeted annual microalbuminura screening, should be intiated for people older than 50 years and with diabetes, hypertension or both as this is shown to be a cost effective approach.^9^,^10^ Since proteinuria is cheap and an effective to screen population at risk of CKD^11^, it should be used more liberally to screen population at risk in Malaysia in view of the strong association with proteinuria in our study.

Other significant laboratory markers found were hyperkalaemia and anaemia. Anaemia is highly prevalent among older people and associated with depression and impaired physical and cognitive function. Anaemia in CKD is associated with reduced quality of life and increased cardiovascular disease, hospitalizations, cognitive impairment, and mortality. As kidney disease progresses, anaemia increases in prevalence, affecting nearly all patients with stage 5 CKD.^12^ The aging process may have an inflammatory component, and some clinical markers of inflammation-associated anaemia overlap with those of iron deficiency. The role of inflammation in CKD-associated anaemia remains poorly understood.

Hyperkalaemia is a common feature of CKD, usually associated with impaired potassium homeostasis. Recent experimental observations suggest that the increase in extracellular potassium directly stimulate potassium excretion through an effect that may be independant or additive to aldosterone.^13^

The referred patients also have higher body weight as compared to the control, and this couple with the diabetic CKD suggest the presence of metabolic syndrome in our patients. Study done in Korea has successfully predicted the occurrence of CKD with increasing body weight.^14^ Despite the strong association of metabolic syndrome with CKD, a causal relationship has not been proven. Studies show that patients with metabolic syndrome have a 2.5-fold higher risk of developing CKD. The risk of microalbuminuria is also increased two-folds in the metabolic synrdrome.^14^

From our study, advanced age beyond 55 years, proteinuria, overweight, anaemia and hyperkalamia are the strong characteristics of the referred CKD patients. Less stronger associations were the presence of hypertension and diabetes.

### Limitation of this study

Relatively small sample size. Since this study is a single centre experience study, the results obtained may not be generalized to other nephrology clinic in Malaysia. However we do strongly believe with this study outcome, we can further enhance the pickup rate of CKD in the community by performing appropriate blood and urine testing at the targeted at risk group with considerable cost saving.

## CONCLUSIONS

Most of the referred CKD patients are advanced age with diabetes, hypertension and overweight. Majority of the CKD remained asymptomatic despite in the advance stage of CKD. This strongly calls for cost effective screening for population at risk.
